# Exploiting hot electrons from a plasmon nanohybrid system for the photoelectroreduction of CO_2_

**DOI:** 10.1038/s42004-024-01149-8

**Published:** 2024-03-20

**Authors:** Ananta Dey, Vitor R. Silveira, Robert Bericat Vadell, Andreas Lindblad, Rebecka Lindblad, Vitalii Shtender, Mikaela Görlin, Jacinto Sá

**Affiliations:** 1 Department of Chemistry-Ångström, Physical Chemistry division, Uppsala University, 751 20 Uppsala, Sweden; 2 Department of Physics, Division of X-ray Photon Science, Uppsala University, 751 21 Uppsala, Sweden; 3 Department of Materials Science and Engineering, Division of Applied Materials Science, Uppsala University, 75103 Uppsala, Sweden; 4 Department of Chemistry-Ångström, Structural Chemistry division, Uppsala University, 751 20 Uppsala, Sweden; 5 Institute of Physical Chemistry, Polish Academy of Sciences, Marcina Kasprzaka 44/52, 01-224 Warsaw, Poland

**Keywords:** Photocatalysis, Nanoparticles, Photocatalysis, Electrocatalysis, Catalyst synthesis

## Abstract

Plasmonic materials convert light into hot carriers and heat to mediate catalytic transformation. The participation of hot carriers (photocatalysis) remains a subject of vigorous debate, often argued on the basis that carriers have ultrashort lifetime incompatible with drive photochemical processes. This study utilises plasmon hot electrons directly in the photoelectrocatalytic reduction of CO_2_ to CO via a Ppasmonic nanohybrid. Through the deliberate construction of a plasmonic nanohybrid system comprising NiO/Au/Re^I^(*phen-NH*_*2*_)(CO)_3_Cl (*phen-NH*_*2*_ = 1,10-Phenanthrolin-5-amine) that is unstable above 580 K; it was possible to demonstrate hot electrons are the main culprit in CO_2_ reduction. The engagement of hot electrons in the catalytic process is derived from many approaches that cover the processes in real-time, from ultrafast charge generation and separation to catalysis occurring on the minute scale. Unbiased in situ FTIR spectroscopy confirmed the stepwise reduction of the catalytic system. This, coupled with the low thermal stability of the Re^I^(*phen-NH*_*2*_)(CO)_3_Cl complex, explicitly establishes plasmonic hot carriers as the primary contributors to the process. Therefore, mediating catalytic reactions by plasmon hot carriers is feasible and holds promise for further exploration. Plasmonic nanohybrid systems can leverage plasmon’s unique photophysics and capabilities because they expedite the carrier’s lifetime.

## Abstract (web summary)

There are numerous examples of plasmonic photothermal catalytic reactions, but the participation of hot carriers remains a subject of vigorous debate. Here, the authors construct a plasmonic nanohybrid system that is unstable above 580  K to show that hot electrons are the main culprit in the photoelectrocatalytic reduction of CO_2_ to CO.

## Introduction

Photocatalysis is a process in which a material called a photocatalyst catalyses a chemical reaction under the influence of light. Photocatalysis is used in a wide range of applications, including air and water purification, the generation of hydrogen fuel, and the degradation of pollutants^1–3^. Classically, light absorption and charge creation rely on materials with a bandgap (in the case of molecular sensitisers, it is the energy difference between the highest occupied energy orbital and the best overlapping lowest unoccupied energy orbital), defining the system’s maximum working voltage. In solid-state systems, excitons are created upon absorption of photons with sufficient energy to overcome the bandgap, leading to electron-hole pairs. Consequently, photons with lower energy than the bandgap energy cannot create excitons, while the excess energy of photons with high energy is lost rapidly as heat. A similar process in molecular catalysts but between the orbital levels mentioned above.

Plasmonic photocatalysis is a new area of research that builds on plasmonics’ unique photophysics to drive chemical processes. Plasmonic materials are metals or metal-like materials that exhibit plasmon resonances, which are collective oscillations of the electrons in the material. These resonances can be excited by light or other electromagnetic radiation and can strongly interact with the electromagnetic field, leading to phenomena such as enhancing specific optical processes. Common examples of plasmonic materials include gold, silver, and copper, widely used in research due to their relatively large and tunable plasmon resonances.

Plasmonic photocatalysis utilises electrical charges formed from the decay of photo-excited plasmon resonance. A plasmon is a quantised oscillation of the electron density, and its decay can generate hot carriers. Hot carriers refer to a non-equilibrium distribution of electrical charges that Fermi-Dirac statistics cannot describe, i.e. hot carriers’ effective temperature is higher than the temperature derived by the Fermi-Dirac occupation probability^4^. Note that the hot carrier temperature is not the temperature in a strict sense; it is only a simplified description of the non-equilibrium distribution. These hot carriers can then be used to generate electrical current or to drive chemical reactions. Hot carrier generation in plasmonics is a relatively new area of research, and it has the potential to be helpful in a wide range of applications, including photovoltaics, energy conversion, and sensing. The energy distribution of hot carriers in plasmonics can depend on various factors, such as the material properties of the metal, the wavelength and intensity of the incident light, and the geometry of the plasmonic structure.

In general, the hot carrier energy distribution is broad, with a fraction of the electrons having energies above the Fermi level of the metal^5,6^. The hot carriers’ ultrafast energy relaxation^7–11^ diminished the high expectations for a plasmonic revolution in applications, making it the bottleneck for photocatalysis to the extent that their involvement of hot carriers remains highly disputed. For example, ref. ^12^ derived a self-consistent theory of the steady-state electron distribution of a metal under continuous wave illumination, which treats thermal and non-thermal effects together. They concluded that the faster chemistry reported is unlikely to originate from high-energy non-thermal electrons. The conclusion was corroborated by ref. ^13^, highlighting that a plasmon-driven process involving the illumination of many nanoparticles will consistently have underestimated photothermal effects that could lead to erroneous interpretations. Despite the significant progress, it remains challenging to disentangle hot carrier catalysis from photothermal effects^14–17^. Nevertheless, authors advocating the use of hot carriers reported their participation in processes such as solar to chemical energy reactions^18–24^, epoxidations^25,26^, dehydrogenations^27^, ammonia electrosynthesis^28^, photoredox catalysis^29–35^, etc.

The hot carriers’ ultrafast relaxation makes their direct use challenging. Therefore, strategies to expedite hot carriers’ lifetimes are being researched, with the most promising being their transfer to suitable acceptors consecutively^36,37^, or simultaneously^38^. These are frequently designated nanohybrid structures, a conceptual framework underpinning this contribution’s scientific essence. The goal is to showcase the direct participation of hot carriers in the catalytic process. A conclusive demonstration of plasmon hot carriers’ mediation in photocatalytic processes could serve as a unifying factor within the plasmon community and catalyse additional exploration and advancement in a range of transformations with profound societal implications.

Herein, a plasmonic nanohybrid system consisting of NiO/Au/Re^I^(*phen-NH*_*2*_)(CO)_3_Cl (*phen-NH*_*2*_ = 1,10-Phenanthrolin-5-amine) was assembled and tested in CO_2_ photoelectroreduction. NiO-sensitised photocathodes are a fast-emerging type of dye-sensitised semiconductors and represent potentially valuable low-cost devices for solar fuel production when coupled with a hydrogen-evolving and CO_2_-reduction catalyst^39–41^. The rhenium complex is a mimic of the catalyst reported by Lehn et al. in 1984, able to catalyse the reduction of CO_2_ to CO on a glassy carbon electrode in a 9:1 DMF/H_2_O (DMF = dimethylformamide) solution during a 14 h experiment with 98% Faradaic efficiency and no significant decrease in performance from catalyst degradation^42–44^. Confirmation of the transfer of plasmon hot carriers to the acceptors was achieved through ultrafast spectroscopy. The reduction of CO_2_ to form CO was verified through photoelectrocatalytic experiments, complemented by mass spectrometry product analysis and unbiased in situ Fourier-transform infra-red spectroscopy (FTIR). These measurements, coupled with the observed low thermal stability of the Re complex, provided substantial support for the hypothesis that hot electrons are the primary contributors to photocatalysis. While a synergistic effect between thermal and hot carriers, given the enhanced hot electron generation at higher temperatures due to phonon coupling^45^, cannot be discounted^46^, the dominant influence in the proposed reaction is attributed to hot carriers, not thermal effects. This suggests that hot carriers can play a pivotal role in catalytic processes when efficiently transferred to suitable acceptors before thermal relaxation.

## Results and discussion

The core premise of this contribution is to validate the direct participation of plasmon-generated hot carriers in a photocatalytic process. Plasmon hot carriers undergo rapid relaxation, resulting in notable local heat generation. Choosing a catalytic process where hot carriers and heat do not mediate equally is crucial. In simpler terms, an endothermic reaction with a substantial enthalpy difference (ΔΗ) is required.

1 $${{{{{\mathrm{C}}}}}}{{{{{{\mathrm{O}}}}}}}_{2}+2{{{{{{\mathrm{H}}}}}}}^{+}+2{{{{{{\mathrm{e}}}}}}}^{-}\longrightarrow {{{{{{\mathrm{CO}}}}}}}+{{{{{{\mathrm{H}}}}}}}_{2}{{{{{\mathrm{O}}}}}}\,\left(-0.53{{{{{\mathrm{V}}}}}}\,{{{{{{\mathrm{vs}}}}}}}\,{{{{{{\mathrm{NHE}}}}}}}\right)$$

CO_2_ conversion to CO (Eq. 1) is an endothermic reaction with an Δ*Η*^o^ = 41 kJ/mol ^47^ (equivalent to a temperature of 4930 K or 0.425 eV). The temperature is significantly higher than one can generate at the plasmon surface^48^. Still, plasmons’ hot electrons have higher energies than required since they inject readily into semiconductors with Schottky barriers higher than 1.0 eV^37^, even if the hole is behind transferred simultaneously^38^. Generally speaking, thermal conversion of CO_2_ to CO typically requires high temperature, high pressure and a catalyst. The exact temperature required for this reaction would depend on the specific process and the reaction conditions. Still, according to ref. ^49^, The CO_2_ to CO reaction only starts at temperatures above 873 K, even when the Boudouard process was used (i.e. CO_2_ + C → 2CO), which offers the lowest temperatures for the conversion. This consideration provides a strategy to circumvent the engagement of the thermal process by crafting a nanohybrid system featuring a catalytic site that decomposes well before the initiation of the thermal process.

The direct electrochemical CO_2_ reduction conversion to CO via sequential one-electron steps involves going through the high-energy radical anion intermediate (CO_2_^•-^), demanding potentials higher than −1.656 V vs SCE at pH 7^50,51^. Therefore, evidence of CO_2_ reduction in organic solvents under unbiased conditions, would be ascribed to hot electrons. Wang et al.^52^ showed that the reaction could be catalysed at room temperature after excitation of multiple localised surface plasmon modes of aluminium nanoparticles with a deep-UV range. Hence, by creating a catalytic site characterised by low thermal stability and substantiating the generation of CO in the absence of electrical bias in organic electrolytes under visible light excitation, it becomes feasible to affirm the engagement of plasmon hot electrons in the photocatalytic process.

**Fig. 1:**
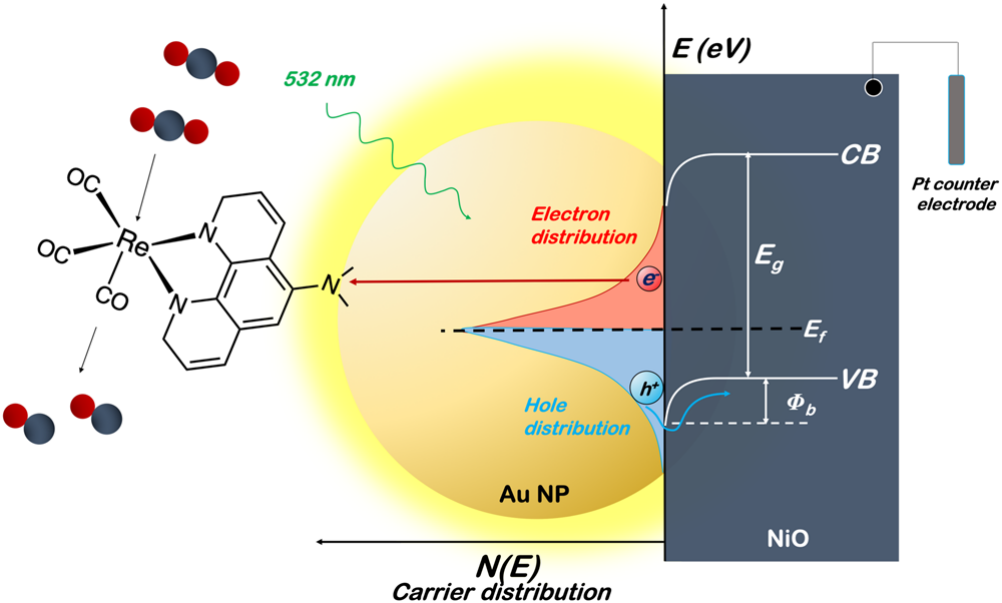
Plasmonic nanohybrid system conserving CO_2_ to CO. Schematic representation of the photoelectrocatalytic system for directly reducing CO_2_ to CO with a plasmon nanohybrid system.

The nanohybrid system was manufactured as a photoelectrode, schematically represented in Fig. 1. The photoelectrode consisted of a hole-accepting material (NiO)^53^, plasmonic material (Au nanoparticles (Au NPs)) and a catalyst (Re^I^(*phen-NH*_*2*_)(CO)_3_Cl) in a geometry consisting of fluorine-doped tin oxide (FTO) conductive glass with a NiO film deposited by screen printing, on which Au NPs were deposited via spray and later selectively functionalised by the Re catalyst through the -NH_2_ groups of the ligand, which in the process loses its protons in accordance to what has been published elsewhere^54^ and will be further on demonstrated. Transition-metal catalysts capable of facile multi-electron transformations offer an alternate pathway to circumvent mechanisms relying on the high-energy radical anion intermediate. Re, Mn, and Ru carbonyl catalysts containing diimine ligands based on 2,2′-bipyridine (bipy) have been investigated extensively for their CO_2_ reduction activity. The first catalyst of this family, the *fac-*Re(bipy)(CO)_3_Cl, was proposed in 1983. Since its discovery as an effective catalyst for CO_2_ reduction, rhenium(I) complexes have been the focus of intense studies^55^.

**Fig. 2:**
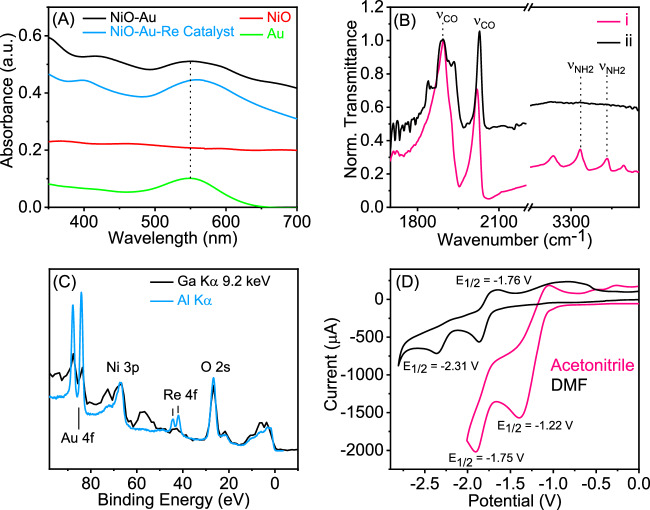
Characterisation of plasmonic nanohybrid system. **A** UV-Vis spectra of the thin films of Au, NiO, NiO-Au, and NiO/Au/Re^I^(*phen-NH*_*2*_)(CO)_3_Cl; **B** (i) FTIR spectra of the Re^I^(*phen-NH*_*2*_)(CO)_3_Cl catalyst powder, (ii) FTIR spectra of the NiO/Au/Re^I^(*phen-NH*_*2*_)(CO)_3_Cl thin film; **C** XPS (Al Kα source) and HAXPES (Ga Kα 9.2  keV source) of the NiO/Au/Re^I^(*phen-NH*_*2*_)(CO)_3_Cl as a film on FTO glass; and **D** Cyclic voltammogram of the Re^I^(*phen-NH*_*2*_)(CO)_3_Cl catalyst in acetonitrile solution and in *N*, *N*-dimethylformamide (DMF) solution using 0.1 M tetrabutylammonium hexafluorophosphate (TBAPF_6_) as supporting electrolyte, by using an Ag/AgCl (0.1 M TBAPF_6_/acetonitrile) reference electrode, glassy carbon as working electrode, and Pt as a counter electrode.

Figure 2A shows the optical spectra of the system components. The *p*-type NiO semiconductor shows a broad and weak absorption across the visible range related to filled trap states. Adding Au NPs yields a new band centred at 560 nm related to the localised surface plasmon absorption. The maximum is slightly shifted to the Au NPs on a thin film, confirming the grafting of the particles to the NiO surface. The Au NPs are 8 ± 2 nm in diameter as determined by dynamic light scattering (DLS), atomic force microscopy (AFM) and scanning electron microscopy (SEM), shown in Figs. S1, S2 and S3, respectively. The average size is consistent with our previous work using the same Au NPs synthesis protocol where the particles were also analysed by transmission electron microscopy (TEM)^45^.

As the catalyst, a mimic of the *fac-*Re(bipy)(CO)_3_Cl, was synthesised, with the difference being that the 2,2′-bipyridine ligand was replaced by the 1,10-Phenanthrolin-5-amine (*phen-NH*_*2*_). The terminal amine group permits direct coordination to the plasmonic gold surface. The attenuated total reflectance Fourier-transformed infra-red (ATR-FTIR) spectrum of the complex is shown in Fig. 2B. In it is visible the carbonyl (1892 and 2019 cm^−1^) and the primary amine stretching (3335 and 3434 cm^−1^). The carbonyl peak at 1892 cm^−1^ has double the intensity of the peak at 2019 cm^−1^, suggesting that the two carbonyl groups are equivalents, which is corroborated by the carbon nuclear magnetic resonance (^13^C NMR) of the complex (Fig. S4). The structure was further confirmed by proton nuclear magnetic resonance (^1^H-NMR) and corroborated by the optical spectrum since this complex class has characteristic absorptions below 400 nm^56^. The complex’s ^1^H-NMR (Fig. S5) and UV-Vis (Fig. S6) spectra can be found in SI.

Adding the catalyst to the NiO/Au film did not significantly change the part of the measurable optical spectrum because the substrate FTO glass and NiO has a sharp absorption edge at 350 nm (Fig. S7), preventing us from detecting the absorption bands of the catalyst. ATR-FTIR confirmed the graphing of the catalyst to Au NPs at the first instance (Fig. 2B) because the amine groups’ vibrational stretching disappeared upon anchoring to the Au NPs. The observation is consistent with the formation of the Au-N bond. N 1*s* X-ray photoelectron spectroscopy (XPS) measurements substantiated this further^57^. In them, the ligand N *1* *s* signal related to the -NH_2_ group of the phenanthroline disappeared once attached to the Au. In contrast, the N *1* *s* from pyridinic groups in the phenanthroline shifted to 399.1 eV (from 398.8 eV) and got broader (FWHM before 1.641 and after attaching 1.812). One expects the N 1*s* from the amine group to shift to lower binding energies as it loses the protons at a rate of about 1 eV per hydrogen atom lost^58^. The observed shift and broadening in the pyridinic N 1*s* were rationalised to be due to the formation of Au-N via the amino groups, which have similar binding energy to the pyridinic groups^57^.

The carbonyl region also changes in peak position and intensity ratio, not total intensity. In the grafted catalyst, all of the carbonyls are different. The peaks are shifted by 10 cm^−1^ to higher wavenumbers (1892, 1933 and 2029 cm^−1^). This suggests some small electron donation from rhenium to the Au NPs since complete one-electron oxidation should shift the values by about 100 cm^−1^^59^. The slight decrease in electron density in the rhenium leads to a breaking of the original equivalency of two of the carbonyls^60^, which resulted in an additional CO vibration peak at 1933 cm^−1^. These observations confirm good electronic connectivity between the catalyst and plasmonic nanoparticles. The decrease in Au absorption peak intensity relates to increased film reflectivity and small heterogeneity.

To further substantiate the anchoring of the molecular catalyst to the Au NPs, XPS analyses were performed using two different excitation sources (Fig. 2C), namely Al Kα (1.48 keV) and Ga Kα (9.2 keV), the latter is commonly referred as hard X-ray photoelectron spectroscopy (HAXPES). The sources’ energy differences enable different penetration probe depths. The analysis with Al Kα shows distinctly the peaks associated with Au and Re 4*f* and the Ni 3*p* and O 2*s*. When the excitation energy source was increased (less surface sensitivity), the signal associated with Au and Re 4*f* reduced dramatically compared to Ni 3*p* and O 2*s*. The result suggests that Au and Re species are collocated as anticipated. The binding energies of Au and Re 4*f*_*7/2*_ were 84.0 eV and 41.9 eV, respectively, characterised by metallic Au^61^ and Re in the +1 state^62^. Ni 3*p*_*3/2*_ has 67.0 eV binding energy between Ni^0^ (65.9 eV)^63^ and Ni^2+^ (69.0 eV for NiO)^64^. The binding energy is close to what has been observed with Ni^2+^ in Ni_2_Ta (66.3 eV)^65^, suggesting a surface reduction of NiO surface. These states are responsible for the NiO visible absorption and are ascribed to trap states.

Figure 2D shows the catalyst’s cyclic voltammetry (CV) profile in acetonitrile, and DMF performed at 50 mV/s with 0.1 M TBAPF_6_ as a supporting electrolyte. The solvents affect the reduction potential but not the shape of the CV, punctuated by two reduction peaks. The first reduction potential (−1.22 V vs Ag/AgCl in acetonitrile) is ascribed to ligand reduction, and the second at −1.75 V vs Ag/AgCl in acetonitrile is the reduction of the metal centre, consistent with previous studies^42^. Further substantiation to this assignment will be provided when discussing ultrafast laser spectroscopy. For comparison purposes, the *fac-*Re(bipy)(CO)_3_Cl reduction peaks in 0.1 M TBAPF_6_ in acetonitrile are −1.34 and −1.725 V vs SCE^55^. The observed potential shift is consistent with ligand replacement^55^. We opted to use acetonitrile as a solvent based on our practical experience with this solvent and the absence of infra-red absorption from the solvent in the regions of interest. However, Lehn-type catalysts have a shorter charge-separated state lifetime in acetonitrile^66^, reducing their catalytic performance.

**Fig. 3:**
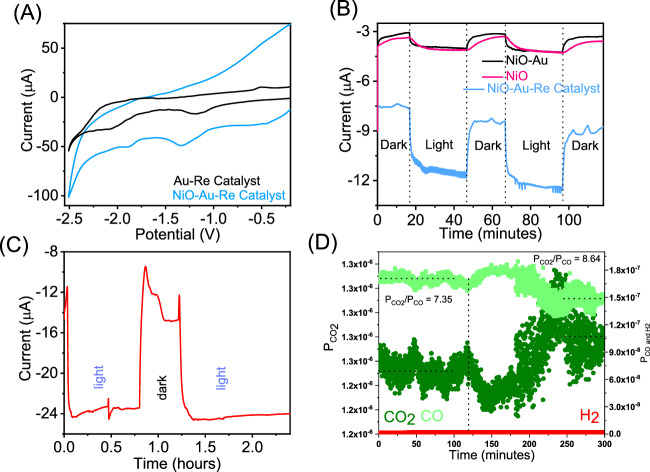
Photoelectrocatalytic performance of the nanohybrid system. **A** Cyclic voltammogram of the Au-Re catalyst and NiO-Au-Re catalyst systems in acetonitrile, using 0.1  M TBAPF_6_ as supporting electrolyte, by using an Ag/AgCl (0.1 M TBAPF_6_/acetonitrile) reference electrode, and Pt as a counter electrode; **B** Chronoamperometry measurement at 295  K on NiO, NiO-Au and NiO-Au-Re catalyst electrodes applying −1.8 V vs Ag/AgCl constant potential and continuous purging with CO_2_ gas in the presence of 0.1  M TBAPF_6_ as supporting electrolyte; **C** Chronoamperometry measurement at 295 K on the NiO-Au-Re catalyst sample (with increase catalyst loading) applying −1.8 V vs Ag/AgCl constant potential and under 20 ml/min CO_2_ gas in the presence of 0.1 M TBAPF_6_ as supporting electrolyte; and **D** mass spectrometry data confirming CO as the product of the photocatalytic process. The light was stopped after 120 min (vertical dashed line), and the CO_2_/CO reverted to 8.6 (fragmentation ratio) after 220 min, revealing a slow dissolution of the product. Note that no significant amount of H_2_ (potential by-product) was detected.

Adding the catalyst to the Au NPs (Fig. 3A) shifted the reduction peaks to −1.15 and −2.05 V vs Ag/AgCl in acetonitrile, suggesting more straightforward ligand reduction. At the same time, reducing the metal centre is more challenging, corroborating the ATR-FTIR that suggested electron donation from rhenium to Au NPs. When the Au NPs were supported on NiO, the process was reversed (−1.32 and −1.88 V vs Ag/AgCl in acetonitrile), suggesting that NiO also donates electron density to Au NPs, thus competing with molecular catalyst donation. Upon adding CO_2_ to the solution, a catalytic wave was observed as soon as the metal was reduced (Fig. S8). The potential reaction mechanism will be discussed later on.

Chronoamperometry measurements during light on/light off measurements are shown in Fig. 3B at −1.8 V vs Ag/AgCl in acetonitrile. Noticeably, the photocurrent becomes detectable after −1.7 V vs Ag/AgCl in acetonitrile. Using the integrated photocurrent and knowing the Re amount (0.18 wt%, determined by ICP-OES), it was possible to calculate the number of catalytic cycles per hour and the turnover frequency (TOF) at −1.8 V vs Ag/AgCl in acetonitrile. Under low CO_2_ solution saturation, the catalyst TOF was 0.156 min^−1^. An average of ten catalytic cycles per hour due to illumination were recorded coupled to the common catalyst deactivation after a 3 h reaction. This is consistent with the catalytic system, not stoichiometric. The quantum yield was estimated to be 0.2%, assuming that each photon creates an electron-hole pair, which is not a perfect depiction of the plasmon process.

Examining catalytic performance parameters under constrained conditions concerning catalyst and CO_2_ amounts allows us to affirm the catalytic nature of the nanohybrid system. However, detecting photocurrent and, more importantly, product formation via quadrupole mass spectrometry (QMS) proved challenging. Consequently, comparable experiments were conducted, employing 1.5 times more catalyst on Au NPs and a heightened CO_2_ flow (20 mL/min). Before delving into the catalytic behaviour under the new conditions, it is noteworthy that the catalyst amount was kept deliberately low to prevent charge transfer between catalyst molecules^67^. Furthermore, the amount of CO_2_ one can flow is capped at 20 mL/min, so one can utilise the most sensitive detector in the QMS, and all the exhaust gas can be analysed. The new conditions resulted in a six-fold measured photocurrent (Fig. 3C). One was able to detect a decrease in CO_2_ (m/z 44)/CO (m/z 28) ratio in the QMS when light was on (Fig. 3D) from 8.64 to 7.35, indicating formation of CO. Note that CO_2_ fragments into CO by about 8–10% depending on the QMS settings. Therefore, it is not prudent to report the amount of CO form. Instead, one should represent the CO/CO_2_ ratio.

It is worth mentioning that the experiments were performed in dry acetonitrile, which limits catalytic performance since protons are needed to extract the O-atom from the CO_2_ molecule. However, the use of dry acetonitrile reduced the chance for hydrogen evolution, a potentially undesirable by-product, as shown in Fig. 3D. It is conceivable that a small amount of water will get dissolved in the solvent during the charging of the chemicals into the reactor. These water molecules play a critical role by quick-starting the reactions at both electrodes. As the reaction proceeds, water is formed at the working electrode and consumed at the counter electrode. The continuous bubbling with dry gas should reduce the chance for additional water to enter the reactor. Note that CO_2_ activation can occur via C–O bond cleavage. However, this was never reported as a possible reaction pathway with Lehn-type molecular catalysts and thus has been omitted as a potential reaction pathway in the presented. What this means is that under the proposed reaction mechanisms, protons are necessary, which was further supported by an increase in the CV catalytic wave current when a more potent proton donor (i.e. 1,1,1,3,3,3-Hexafluoro-2-propanol (HFIP), was introduced (Fig. S9).

The impact of illumination was most evident in the generated current rather than the overpotential, with only approximately a 50 mV shift detected. Consequently, it prompts a reasonable enquiry into whether these changes stem from hot electrons or the heat generated through plasmon thermalisation. Additional support for hot electron involvement can be drawn from catalytic data. Firstly, the average temperature in the solution exhibited minimal change, staying within a 20 K range even under vigorous stirring. Secondly, the catalyst decomposed below 578 K, constraining the usable thermal window. Thirdly, the current exhibited an instantaneous response to light, aligning with expectations if hot carriers were involved^68^. Lastly, the photosystem lacking NiO demonstrated inferior performance compared to the complete system, contradicting expectations if heat were the sole factor for enhanced catalysis. This discrepancy arises because NiO’s role is to extract holes, thereby reducing recombination between electrons and holes, which would otherwise result in heat formation. This does not mean that heat cannot have some minor positive contribution to the catalytic yield because small temperature gradients can induce convection, and surface heating can affect reaction rates.

**Fig. 4:**
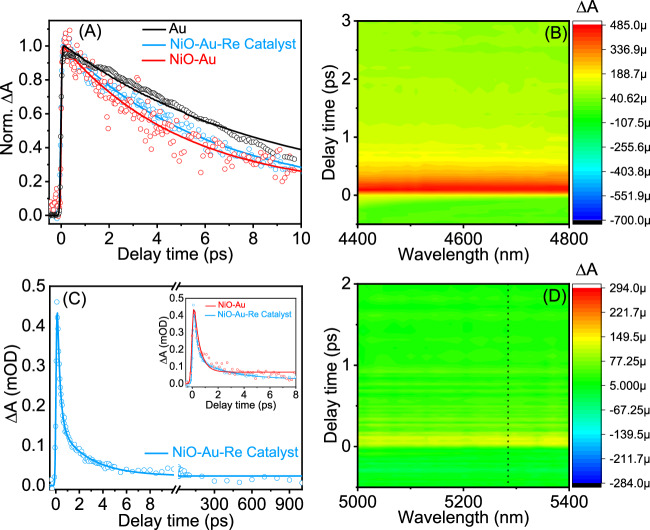
Plasmonic hot carrier ultrafast dynamics. **A** TAS kinetic traces extracted at 470–485  nm (maximum of the high-energy positive band) exciting the Au NPs plasmon at 550 nm; **B** TIRAS contour plot of NiO/Au/Re^I^(*phen-NH*_*2*_)(CO)_3_Cl after excitation of the Au NPs plasmon at 550  nm; **C** TIRAS kinetic trace NiO/Au/Re^I^(*phen-NH*_*2*_)(CO)_3_Cl extracted at 4500 nm exciting the Au NPs plasmon at 550  nm; and **D** TIRAS contour plot of Au/Re^I^(*phen-NH*_*2*_)(CO)_3_Cl in the infra-red carbonyl region after excitation of the Au NPs plasmon at 550 nm. The dotted line refers to the position of the carbonyl band located at 1892 cm^−1^.

Yet, establishing more robust supporting evidence for hot carrier involvement is crucial. To achieve this, a series of in situ studies were conducted. As the overall process involves catalytic reduction, providing evidence of the nanohybrid system’s reduction without external potential would substantiate the role of hot electrons. Unbiased ultrafast transient absorption spectroscopies were employed to scrutinise the participation of hot electrons in catalysis. Transient absorption spectroscopy (TAS) facilitates the monitoring of plasmon resonance (Fig. 4A). Analyzing the kinetics of the rapid signal decay allows for the extraction of the electron-phonon (e-ph) lifetime, a parameter highly sensitive to the number of carriers in resonance^36^. The e-ph for Au nanoparticles on glass was estimated to be 8.5 ± 1.2 ps.

The e-ph process starts after the electron-electron (e-e) scattering process is found to happen less than 100 fs (shorter than the instrument response function), calculated from the TAS rising edge function that, and as expected^69,70^. Link and El-Sayed showed that e-ph of Au nanoparticles around 15 nm in size in solution had an e-ph of 3–4 ps but increases with increased laser fluence^71^ and changes in a dielectric medium^72,73^. Since the measurements were performed on low-density Au NPs films to avoid intraparticle interference, the laser fluency was on the high end of what Link & El-Sayed used to ensure good signal-to-noise data. Moreover, the samples were measured on solid substrates and a different dielectric medium than in solution, furthering the increase in e-ph lifetime. Furthermore, the Au NPs were excited at 550 nm on the red side of the plasmon absorption peak to ensure that only the plasmon was excited, i.e. to avoid intraband excitation. Considering all these factors, the estimated e-ph is within reason of what has been observed. Critically, the TAS of all the samples were performed under the same laser fluency and dielectric medium to ensure that the extracted e-ph lifetimes could be compared.

Depositing Au on NiO resulted in a decrease of the Au plasmon e-ph to 5.1 ± 0.6 ps. This is consistent with hole transfer from Au to NiO since the process increases the number of electrons in the resonance, which lowers the average temperature of the electrons in the resonance^36^. Similarly, adding the catalyst to Au NPs reduced the Au plasmon e-ph lifetime to 6.1 ± 0.4 ps. Plasmon resonance relates to particle morphology. However, the dynamic changes in the resonance are connected to the number of electrons participating in the resonance and their energy (or electron temperature). An electron acceptor (like the catalyst) reduces the resonance lifetime by taking hot electrons from it. Therefore, the observed change is a combination of two factors that affect the resonance in an antagonist way, namely, (i) taking electrons from the resonance should increase the e-ph lifetime since fewer electrons share resonance energy, but (ii) the electrons are removed hot (with higher kinetic energy) that reduces the average electron temperature that remains in the resonance and consequently e-ph lifetime^31^. The complete system (NiO/Au/Re^I^(*phen-NH*_*2*_)(CO)_3_Cl) has an e-ph lifetime of 7.2 ± 0.6 ps, which is consistent with a reduction of e-ph due to energy (to the catalyst) and hole (to NiO) transfer that is slightly counteracted by the electron transfer to the catalyst.

To further substantiate the claim that holes and electrons are transferred to specific acceptors, unbiased transient infra-red absorption spectroscopy (TIRAS) measurements were performed. TIRAS is highly sensitive to free carriers and changes in vibrations^74^. TIRAS of Au on NiO revealed a broad featureless absorption (similar to the TIRAS contour plot of the complete system in Fig. 4B), characteristic of forming a quasi-metallic state due to free carriers^32^. The hole injection time from the rising edge was 196 ± 91 fs. The signal decay was fitted with two exponential components, with the first component (τ_1_ = 0.45 ± 0.2 ps (>90%)) accounting for most of the signal, making it difficult to determine τ_2_ reliably. More importantly, the signal decays completely in less than 1 ns, which means that under continuous illumination, it would be difficult to have charge accumulation necessary to catalyse reactions. The decays are associated with charge recombination at the interface of Au and NiO, similar to NiO-molecular dye hybrid systems^75^. The shorter recombination time relates to charge recombination straight after injection, while the longer is associated with the charge that escaped from the interface to the bulk of NiO.

The complete system saw an even faster injection time of 124 ± 16 fs, as well as a slight increase in carriers’ lifetime (Fig. 4C). The shorter-lived carriers required a fitting with two components τ_1_ = 0.25 ± 0.05 ps (76%) and τ_2_ = 2.57 ± 0.61 ps (20%), indicative that the addition of the catalyst prolongs the lifetime of the holes transferred to NiO since the catalyst extracts the electrons, effectively restoring Au NPs neutrality. Moreover, Fig. 4C reveals that 4% of the charges survive more than 1 ns, making them available for chemical reactions. Note that the TIRAS dynamics are without applied potential that will further expedite the charge-separated state lifetime and thus increase the amount of charge available for reaction.

One reason for emulating Lehn’s catalyst is the remarkable sensitivity of the infra-red-active carbonyl (C-O) stretching to electronic variations at the metal site. This characteristic renders it a perfect tool for examining alterations in the metal oxidation state^76^. The transformation of CO_2_ to CO using a Re-carbonyl catalyst involves a two-electron stepwise reduction, with the initial reduction targeting the ligand^77^. Notably, alterations in the carbonyl stretching region are not anticipated in the unbiased ultrafast TIRAS since only the first reduction step (i.e. ligand reduction) is detectable at this rapid time scale^60^. This is precisely the case; namely, the complete systems had no changes in the Re-carbonyl region (Fig. S10). However, the system had a broad featureless signal related to free carriers, suggesting that the first electron transfer is to the ligand.

To further substantiate this hypothesis, the TIRAS of the Au-Re catalyst without NiO was measured (Fig. 4D). The rationale behind this experiment was to minimise signal interference from hot holes in NiO. Despite the challenge of low signal-to-noise, when the Au-Re catalyst was excited to the red of the maximum plasmon absorption (to avoid intraband excitation), it produced a broad and featureless signal with positive absorption. Given the absence of a semiconductor and the fact that plasmon excitation energy prevented intraband excitation, it can be concluded that the signal is linked to the injected electron in the catalyst. By analyzing CV data and observing no changes in the Re-carbonyl region, it can be inferred that the signal corresponds to the electron reduction of the phenanthroline ligand. The signal’s shape implies that the electron is distributed across the aromatic rings, behaving akin to a free carrier.

Having established that plasmon-induced hot electrons reduced the linker through ultrafast spectroscopy, the subsequent step involved substantiating the second reduction leading to CO formation. This verification was done through in situ unbiased Fourier-transformed infra-red (FTIR) spectroscopy experiments conducted under 532 nm illumination. These experiments were executed in acetonitrile saturated with CO_2_ and argon.

**Fig. 5:**
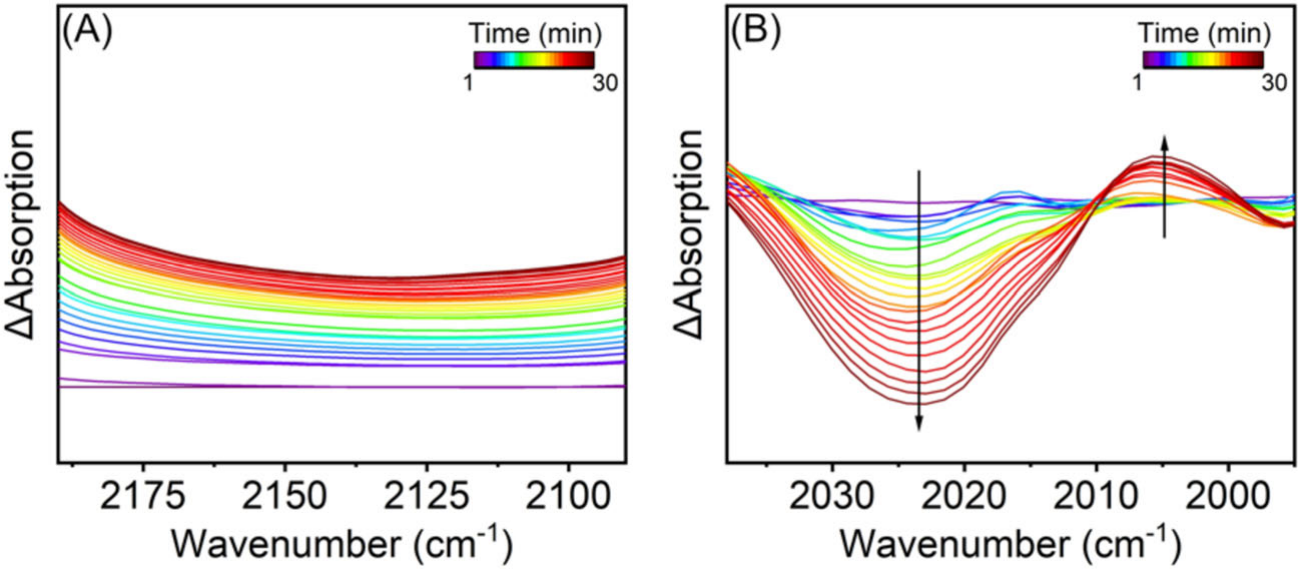
In situ vibrational study depicting CO_2_ reduction. In situ unbiased FTIR experiments in acetonitrile illuminated with 532 nm CW laser. Temporal evolution (30 min) of the FTIR spectra under illumination subtracted by the spectrum collected on the dark **A** outside the region of interest depicting the shift of the baseline due to free carriers, and **B** of the carbonyl region unaffected by the acetonitrile absorption bands upon baseline correction to remove free carriers contribution.

Figure 5A shows the temporal evolution of the FTIR signal outside the region of interest (carbonyl region 2100–1900 cm^−1^), which reveals a background shift with increased illumination. A similar transient behaviour was detected in argon but not visible when the system was not irradiated, confirming that this signal is related to the effect of light not reacting to the atmosphere. This light-dependent background shift is ascribed to free carriers^75,78^, in the NiO and ligand, corroborating the ultrafast transient data.

The background shift due to free carriers makes data analysis challenging. The change was correct, fitting a baseline with the same parameters. Subtracting the spectra from the spectrum collected in the dark enabled analysis of the temporal evolution of the carbonyl groups not affected by the acetonitrile absorption. Unfortunately, acetonitrile has strong absorption bands between 1800 and 1900 cm^−1^, making it challenging to follow the equivalent carbonyl peaks. Therefore, the analysis was restricted to the inequivalent carbonyl centred at 2024 cm^−1^ (ATR-FTIR 2019 cm^−1^). Solvation effects can justify the slight difference^79^.

It is evident that under illumination, the 2024 cm^−1^ peak decreases in intensity and a new peak appears at 2005 cm^−1^. The reduction in the band centred at 2024 cm^−1^ related to the inequivalent carbonyl is correlated with ligand reduction because this was, to some extent, observed in the sample illuminated in argon. The ligand reduction induces a structural change in the catalyst complex, making all the CO originally coordinated to the Re^+^ centre equivalent. Still, we need additional support for this hypothesis. The possibility of ligand detachment was discarded because one did not free CO due to the absence of its characteristic band at 2193 cm^−1^ in the spectra, and the dry catalyst FTIR signal before and after illumination in argon was the same. Klein et al.^80^ proposed a similar in the catalyst with a bipy ligand. According to them, the ligand plays a critical “noninnocent” role by storing the first reducing equivalent in a Re^I^(bipy^•-^) state, leading to a complex reorganisation. The appearance of the peak at 2005 cm^−1^ seems consistent with the formation of a new Re^I^-CO species since the finding was only visible when the sample was illuminated and was in the presence of CO_2_, which is consistent with what ref. ^77^ proposed. Their mechanism suggests the formation of such species after two proton additions and the liberation of H_2_O. Both observations suggest catalyst reduction and the appearance of CO without applied potential, consistent with a mechanism involving hot carriers.

**Fig. 6:**
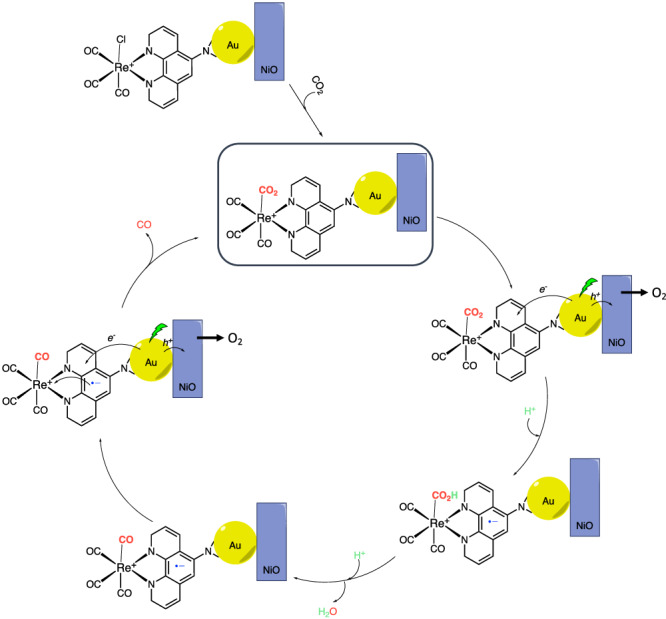
Reaction mechanism. A schematic representation of the proposed reaction mechanism depicts the sequential reduction and protonation of the substrate until the release of the CO product.

Considering the catalytic and characterisation data, we proposed a catalytic mechanism depicted in Fig. 6. The initial step is the coordination of CO_2_ to the catalyst by exchange with the Cl^−^ group after an initial 2*e*^*−*^ reduction, consistent with previously reported^81,82^, which can be performed with hot electrons or external bias. Excitation of Au NPs LSPR forms hot electrons and holes. The hot holes are injected into the NiO and reacted on the counter electrode, producing O_2_. The first electron reduces the ligand, consistent with the TIRAS measurements and the most updated mechanism proposals for Lehn’s catalyst^77^. After this, the bonded CO_2_ molecule undergoes two sequential protonations, forming a new CO species bonded to Re^+^ (confirmed by unbiased in situ FTIR) and liberating one H_2_O molecule. According to Rotundo et al., the second protonation is slightly endothermic^77^, partially explaining that the newly formed Re^I^-CO species FTIR band is not very intense after 30 min on stream. This is further supported by an increase in the CV catalytic wave current when a more potent proton donor (HFIP), was introduced (Fig. S9). The CO molecule is released with the second reduction by the plasmon hot electrons, regenerating the catalyst and enabling the binding of a new CO_2_ molecule.

## Conclusion

In summary, our study establishes the role of plasmon hot carriers in the photoelectrochemical reduction of CO_2_ at room temperature. Our unique approach involves anchoring a molecular catalyst with moderate thermal stability to the plasmonic Au surface. This strategy allows for the selective extraction of hot electrons, enabling catalytic conversion while minimising the contribution of the photothermal process. Integrating photoelectrocatalysis, electrochemistry, and advanced spectroscopy provides conclusive evidence of hot carriers transferring to accepting units and actively participating in the catalytic cycle. The reaction mechanism unveiled a sequential reduction of the rhenium complex with two protonations between these reduction steps. Our findings suggest that the second protonation, leading to the liberation of water molecules, constitutes the rate-limiting step in the process. This resolution settles a long-disputed hypothesis regarding the involvement of plasmon hot carriers in catalysis. To harness the potential of hot carriers in catalysis, it is imperative to engineer plasmonic materials to facilitate the rapid transfer of hot carriers, within hundreds of femtoseconds, to suitable accepting units possessing appropriate energy levels for the desired catalytic transformation. The ultrafast relaxation of hot carriers on the plasmonic material surface poses a challenge, necessitating careful design to optimise their utilisation in catalytic processes.

## Methods

### Sample preparation

#### Au nanoparticle (NPs) synthesis

Sodium citrate tribasic dihydrate 50 mL (6.6 mM) water solution was taken in a 100 mL round bottom flask and stirred at 70 ^o^C in an oil bath. Then, 0.1 mL (2.5 mM) tannic acid was added to the reaction mixture. Finally, 1 mL of (25 mM) HAuCl_4_ was added instantly. After 5 min, the colour of the reaction mixture changed from dark blue to wine. The colour change confirms the formation of the Au nanoparticles. The synthesised Au nanoparticles were stored in a fridge. The size of the Au nanoparticles was analysed using dynamic light scattering (DLS).

#### Re^I^(phen-NH_2_)(CO)_3_Cl (phen-NH_2_ = 1, 10-Phenanthrolin-5-amine) catalyst synthesis

1, 10-Phenanthrolin-5-amine 200 mg (1.02 mmol) and pentacarbonylchlororhenium (I) 368.94 mg (1.02 mmol) were suspended in 40 mL toluene using a 100 mL round bottom flask. The reaction mixture was stirred at 110 ^o^C for 6 h. Then, the reaction mixture was cooled down to room temperature. The obtained yellow colour precipitate was filtered using a G4 gooch crucible under vacuum and finally washed three times with 50 mL toluene. Yield 379.57 mg (74%). ^1^H-NMR (400 MHz, DMSO-*d*_*6*_): δ ppm 9.38 (1H, dd, *J* = 5.2, 1.2 Hz), 9.12 (1H, dd, *J* = 8.8, 1.2 Hz), 8.94 (1H, dd, *J* = 4.8, 1.2 Hz), 8.49 (1H, dd, *J* = 8.4, 1.2 Hz), 8.06 (1H, dd, *J* = 8.6, 5.2 Hz), 7.79 (1H, dd, *J* = 8.4, 5.2 Hz), 7.07 (1H, s), 6.89 (2H, s). ^13^C NMR (100 MHz, DMSO-*d*_*6*_): δ(ppm) 197.90, 190.18, 153.15, 147.87, 146.73, 144.88, 139.62, 135.60, 134.35, 132.68, 126.21, 125.04, 123.56, 101.17.

#### Preparation of the thin films

The preparation and process of NiO film:

The NiO paste was purchased from Solaronix and used as received. A small portion of the NiO paste was placed on a screen, placed on top of the cleaned FTO glass, and manually printed on the conducting side of the FTO glass. Then, the NiO-printed FTO glass plates were annealed at 500 ^o^C for 1 h at a rate of 10 ^o^C/min.

#### Assembly of the Au nanoparticles on NiO film

The synthesised Au nanoparticles were sprayed manually on NiO films and annealed at 500 ^o^C for 1 h at a rate of 10 ^o^C/min.

#### Assembly of the catalyst on the NiO/Au surface

We prepared a complete system with two different loadings of catalysts, which served other purposes in our study.

##### Lower catalyst loading:

The annealed NiO-Au films were dipped into a 2 mg/mL solution of the synthesised Re^I^(*phen*-*NH*_*2*_)(CO)_3_Cl catalyst in Dimethylformamide for 48 h. Finally, we could get the self-assembled NiO/Au/Re^I^(*phen-NH*_*2*_)(CO)_3_Cl composite system. We have also sprayed Au nanoparticles only on FTO glass for experimental purposes.

##### Higher catalyst loading

The annealed NiO-Au films were dipped into a 2 mg/mL of the synthesised Re^I^(*phen*-*NH*_*2*_)(CO)_3_Cl catalyst in Dimethylformamide for 48 h at 100 ^o^C temperature. Finally, we could get the self-assembled NiO/Au/Re^I^(*phen-NH*_*2*_)(CO)_3_Cl composite system. We have also sprayed Au nanoparticles only on FTO glass for experimental purposes.

### Samples characterisation

#### UV-Vis measurements

The UV-Vis spectra were collected using a Cary 5000 UV-VIS-NIR spectrophotometer.

#### DLS measurements

The DLS data were collected in a Malvern Zetasizer nanoS instrument, and a total of three measurements comprised of 12 scans each time was done.

#### Atomic force microscopy (AFM) measurements

The AFM data was collected on AFM nanosurf with a long Si cantilever in tapping mode with Al reflex.

#### ^*1*^*H and*^*13*^*C nuclear magnetic resonance (NMR) measurement*

The NMR were measured using a JEOL 400 machine in DMSO-*d*_*6*_.

#### Standard Fourier-transformed infra-red (FTIR) measurements

FTIR data were measured using a universal ATR sampling assembly on a VERTEX 70 v instrument.

#### In situ Fourier-transformed infra-red (FTIR) measurements

FTIR data were measured in transmission in a TensorII instrument. The photosystem films were deposited on CaF_2_ and inserted in a homemade liquid transmission flow cell with adjustable light path length. The samples were measured in dry acetonitrile after saturation for 1 h with Ar or CO_2_. The sample was illuminated with a 532 nm CW laser, and the changes were monitored at minute intervals with a spectral resolution of 4 cm^−1^ with an MCT detector.

#### Electrochemistry measurements

The electrochemical data were measured using an EmStat potentiostat instrument. For the electrochemical experiments, a typical cylindrical closed cell was used. The FTO films were placed on the side of the cell so that it could face the light.

#### Bulk electrolysis

Pt wire counter electrode and Ag/AgCl (0.1 M TBAPF_6_/acetonitrile) reference electrode were purchased from Redox.me and used as received. 0.1 M Tetrabutylammonium hexafluorophosphate purchased from Merck was used as a supporting electrolyte without further purification in dry acetonitrile.

#### Photoelectrocatalytic chronoamperometry

The photoelectrode exposed area to light is 0.79 cm^2^. Plasmonic excitation was performed with a 532 nm laser of 53 mW/cm^2^ intensity. Pt wire counter electrode and Ag/AgCl (0.1 M TBAPF_6_/acetonitrile) reference electrode were purchased from Redox.me and used as received. Tetrabutylammonium hexafluorophosphate purchased from Merck was used as a supporting electrolyte without further purification.

Similar experiments were performed under CO_2_ gas flow (20 mL/min) to enable the detection of the exhaust on a Hiden Analytical quadrupole mass spectrometer.

#### Quantum yield (QY) calculations

Plasmonic hot carriers are formed via resonance decay, not direct photon excitation. This makes quantum yield calculations with plasmonic complex since the photons absorbed are not equivalent to a maximum number of electrons produced (denominator of quantum yield formula), which is the case on gap materials, where one photon can only have one electron-hole pair. Nevertheless, we used the classical formula to calculate quantum yield (QY), namely:

QY = (number of electrons reacted/number of photons absorbed) × 100

#### XPS and HAXPES measurements

Photoelectron spectra were recorded using two-photon energies, Al Kα (1487 eV) and Ga Kα (9.25 keV). The electron energy analyser used was a Scienta Omicron EW-R4000. The spectra were recorded in a vast region between 0 and 100 eV electron binding energy, giving a significant difference in information depth for the two-photon energies. The samples were measured as thin films on conductive glass.

#### Inductively coupled plasma-optical emission spectrometry (ICP-OES) measurement

The ICP-OES was used to estimate the rhenium amount in the sample. The film was digested for four hours in 4 ml of nitric acid (Nitric acid 65%, Fisher Scientific), and the small probe was diluted ten times with Milli-Q water containing 5% HNO_3_ and filtered with 0.2-μm syringe filters (Whatman) before measurement. Avio 200 Scott/Cross-Flow Configuration was used for ICP measurements. A calibration curve was formed for the measurements using a Rhenium Calibration Standard (Merk). Concentrations of 0, 0.1, 1 and 10 ppm of the Re were used to create a four-point linear regression. All measured values are within a relative standard deviation (RSD) of 2%.

#### Transient absorption spectroscopy (TAS)

A 40-fs pulsed laser with a 3 kHz repetition rate was generated through the Libra Ultrafast Amplifier System designed by Coherent. An optical parametric oscillator (TOPAS- prime, Light Conversion) generated the excitation beam. The signals were detected with a UV-NIR detector and a Newport MS260i spectrograph with interchangeable gratings. The fundamental laser (probe, 795 nm) passes through the delay stage (1–2 fs step size) and is focused in a Sapphire optical window to generate visible light from 400 to 750 nm. The instrument response function obtained for our system is ca. 95 fs.

#### Transient infra-red absorption spectroscopy (TIRAS)

A 40-fs pulsed laser with a 3 kHz repetition rate was generated through the Libra Ultrafast Amplifier System designed by Coherent. Two optical parametric oscillators (TOPAS- prime, Light Conversion) created the excitation beam and the probe light in the mid-IR (3000–10000 nm). The signals were detected with a Horiba iHR 320 spectrometer. The pump laser power was constantly monitored with less than a 2% standard deviation. The timing resolution, i.e., the instrument response function, is ca. 100 fs.

### Supplementary information

Supplementary Information

Description of Additional Supplementary Files

Supplementary Data 1

## Data Availability

The data related to the figures in the paper are provided as Excel files in Supplementary Data 1. Additional data supporting this study’s findings are available from the corresponding author upon request.
