# Supplementary material for: Exploiting hot electrons from a plasmon nanohybrid system for the photoelectroreduction of CO2
Source: Commun Chem. 2024 Mar 20;7:59. doi: 10.1038/s42004-024-01149-8 (PMC10954701; doi:10.1038/s42004-024-01149-8)
Supplement: Supplementary file 1 — Supplementary Information [file 42004_2024_1149_MOESM1_ESM.docx]

**Supporting information**

**Exploiting hot electrons from a plasmon nanohybrid system for the photoelectroreduction of CO_2_**

Ananta Dey^1#^, Vitor R. Silveira^1#^, Robert Bericat Vadell^1^, Andreas Lindblad^2^, Rebecka Lindblad^2^, Vitalii Shtender^3^, Mikaela Görlin^4^, Jacinto Sá^1,5^*

# equal contribution

*Corresponding author: jacinto.sa@kemi.uu.se

^1^ Department of Chemistry-Ångström, Physical Chemistry division, Uppsala University, 751 20 Uppsala, Sweden

^2^ Department of Physics, Division of X-ray Photon Science, Uppsala University, 751 21 Uppsala, Sweden

^3^ Department of Materials Science and Engineering, Division of Applied Materials Science, Uppsala University, 75103 Uppsala, Sweden

^4^ Department of Chemistry-Ångström, Structural Chemistry division, Uppsala University, 751 20 Uppsala, Sweden

^5^ Institute of Physical Chemistry, Polish Academy of Sciences, Marcina Kasprzaka 44/52, 01-224 Warsaw, Poland

**Additional data:**

**FIGURE S1:** Dynamic light scattering (DLS) analysis of Au NPs in water.

**
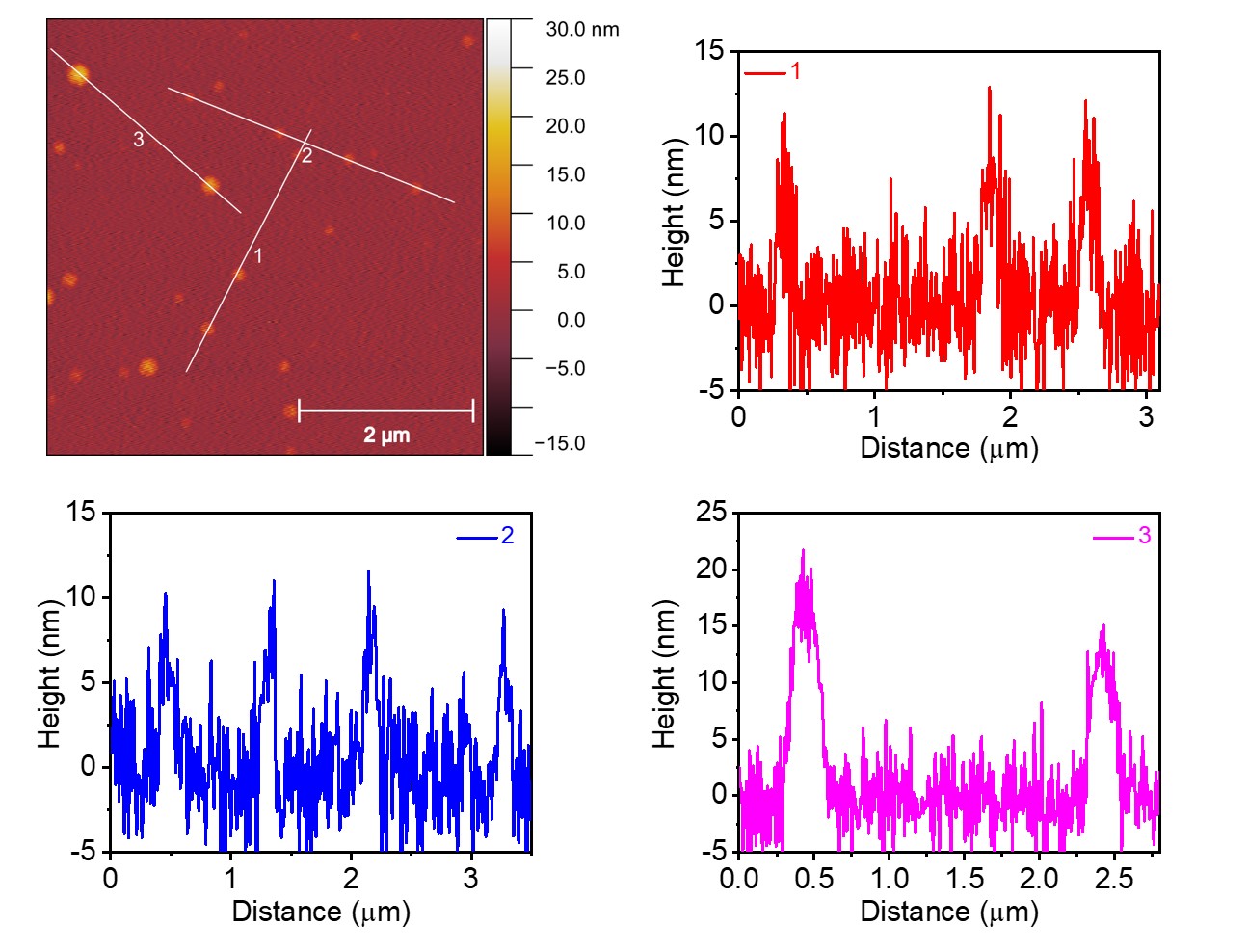
**

C

D

A

B

**FIGURE S2:** AFM of Au NPs on Si after annealing. A) AFM micrograph; and B to D) line traces showing average particle height.

**



FIGURE S3:** SEM of Au NPs on FTO glass with two magnifications. The figures show a uniform dispersion of particles and homogeneity in size ranging from 5-10 nm.


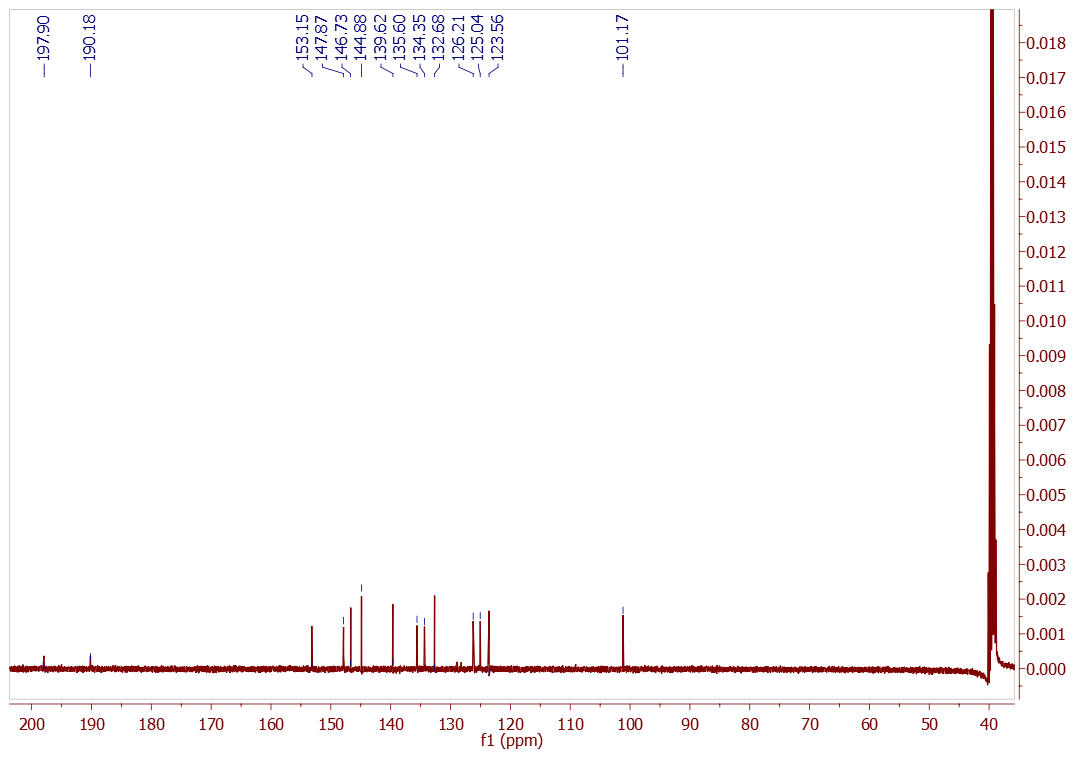


**FIGURE S4:** ^13^C NMR spectrum of Re^I^(*phen-NH_2_*)(CO)_3_Cl in DMSO-*d_6_*.


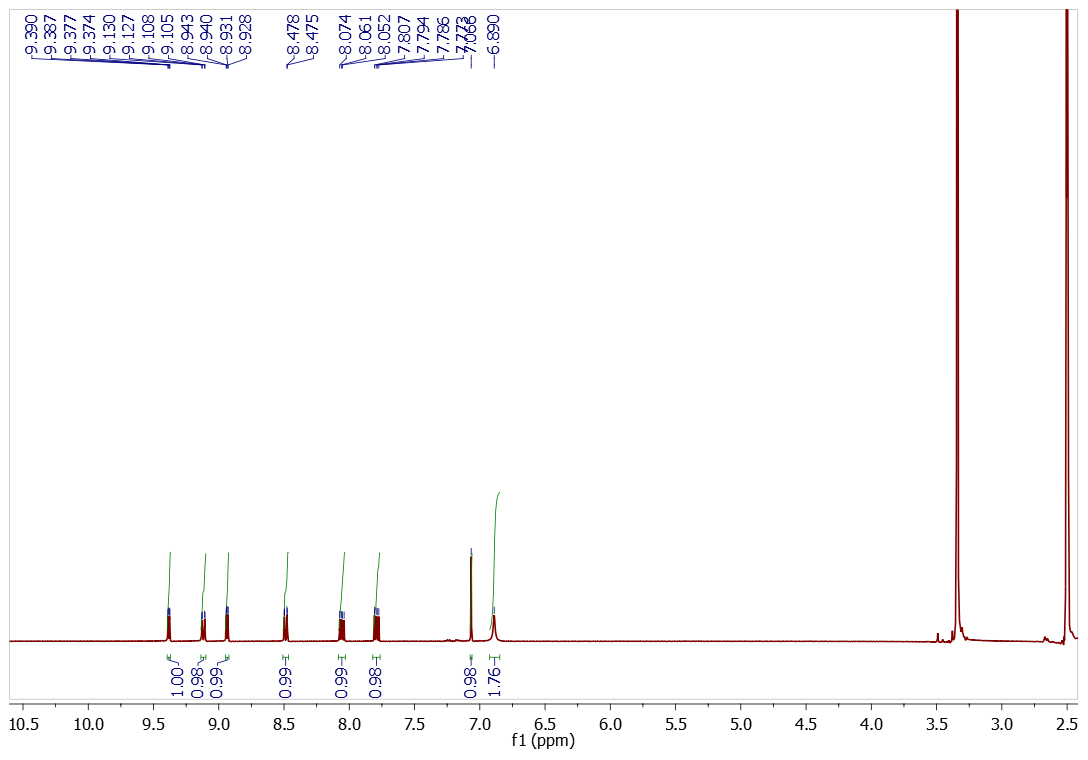


**FIGURE S5:** ^1^H Nuclear magnetic resonance (NMR) spectrum of Re^I^(*phen-NH_2_*)(CO)_3_Cl in DMSO-*d_6_*.

**FIGURE S6:** Optical spectrum of Re^I^(*phen-NH_2_*)(CO)_3_Cl in dimethylformamide.

**
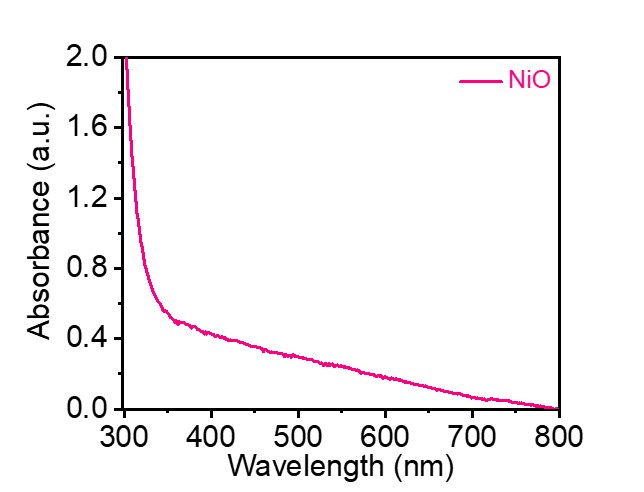
**

**FIGURE S7:** Optical spectrum of NiO on FTO glass around the NiO absorption edge.

**FIGURE S8:** Cyclic voltammograms of the NiO/Au/Re^I^(*phen-NH_2_*)(CO)_3_Cl in argon and CO_2_ purging conditions.


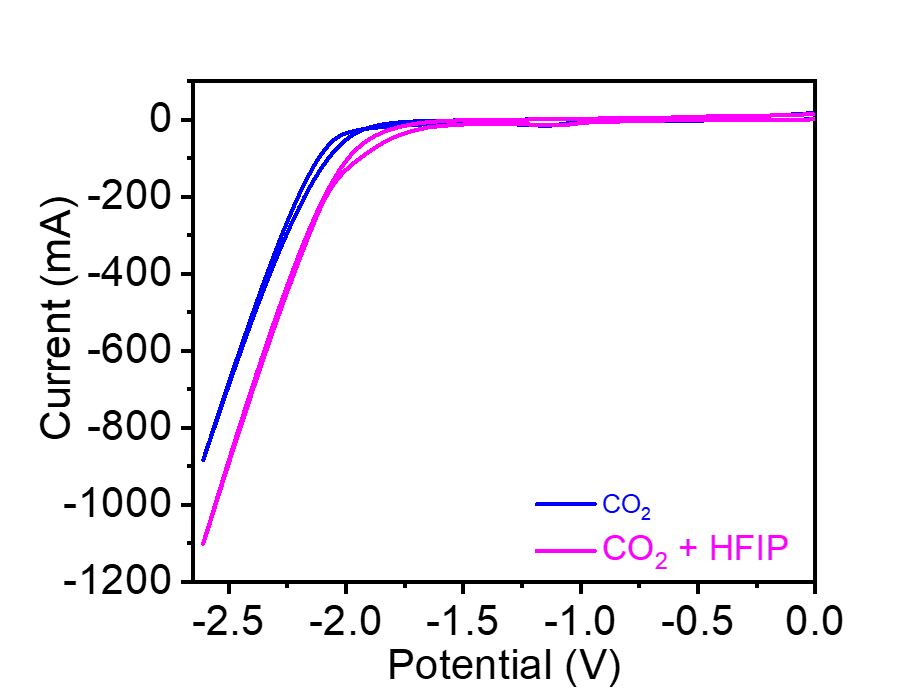
**FIGURE S9:** Cyclic voltammograms compare the NiO/Au/ReI(phenNH2)(CO)3Cl in CO2 after the introduction of a proton donor, 1,1,1,3,3,3-Hexafluoro-2-propanol (HFIP).

**FIGURE S10:** TIRAS contour plot of NiO/Au/Re^I^(*phen-NH_2_*)(CO)_3_Cl in the carbonyl region.

**Data availability:**

The data related to the figures in the paper are provided as excel files in Source data. Additional data supporting this study's findings are available from the corresponding author upon request.
