# Supplementary material for: Exploiting hot electrons from a plasmon nanohybrid system for the photoelectroreduction of CO2
Source: Commun Chem. 2024 Mar 20;7:59. doi: 10.1038/s42004-024-01149-8 (PMC10954701; doi:10.1038/s42004-024-01149-8)
Supplement: Supplementary file 2 — Description of Additional Supplementary Files [file 42004_2024_1149_MOESM2_ESM.pdf]

Description of Additional Supplementary Files

File name: Supplementary Data 1

Description: Excel files of source data underlying the graphs and charts presented in the main figures.
